# Lab-On-A-Chip Device for Yeast Cell Characterization in Low-Conductivity Media Combining Cytometry and Bio-Impedance †

**DOI:** 10.3390/s19153366

**Published:** 2019-07-31

**Authors:** Julien Claudel, Arthur Luiz Alves De Araujo, Mustapha Nadi, Djilali Kourtiche

**Affiliations:** Institut Jean Lamour Lorraine University, French National Center for Scientific Research (CNRS–Unité Mixte de Recherche 7198), 54011 Nancy, France

**Keywords:** biosensor, impedance spectroscopy, microfluidic, single cell, cytometry

## Abstract

This paper proposes a simple approach to optimize the operating frequency band of a lab-on-a-chip based on bio-impedance cytometry for a single cell. It mainly concerns applications in low-conductivity media. Bio-impedance allows for the characterization of low cell concentration or single cells by providing an electrical signature. Thus, it may be necessary to perform impedance measurements up to several tens of megahertz in order to extract the internal cell signature. In the case of single cells, characterization is performed in a very small volume down to 1 pL. At the same time, measured impedances increase from tens of kilo-ohms for physiological liquids up to several mega-ohms for low conductivity media. This is, for example, the case for water analysis. At frequencies above hundreds of kilohertz, parasitic effects, such as coupling capacitances, can prevail over the impedance of the sample and completely short-circuit measurements. To optimize the sensor under these conditions, a complete model of a cytometry device was developed, including parasitic coupling capacitances of the sensor to take into account all the impedances. It appears that it is possible to increase the pass band by optimizing track geometries and placement without changing the sensing area. This assumption was obtained by measuring and comparing electrical properties of yeast cells in a low-conductivity medium (tap water). Decreased coupling capacitance by a factor higher than 10 was obtained compared with a previous non-optimized sensor, which allowed for the impedance measurement of all electrical properties of cells as small as yeast cells in a low-conductivity medium.

## 1. Introduction

For many years now, research has focused on the development of lab-on-a-chip devices for biomedical applications [1] and applications such as the analysis of water quality [2]. These systems may allow for the performance of highly sensitive detections while decreasing cost and size, and improving usability and portability [3]. By reducing the size of the investigated volume, it is possible to significantly increase the sensitivity for the detection of particularly low particle numbers or cell concentrations [4,5]. It is even possible to detect and characterize a single cell or microparticle [6]. 

Bioimpedance measurement, such as bioimpedance spectroscopy (BIS), permits the characterization of physiological properties of cells and bacteria over a large range of applications from human tissues analysis to the detection of bacteria or microorganisms. This technique is widely used as a diagnostic tool [7] or for medical imaging [8]. However, it remains difficult to use in the case of low concentration samples due to the low levels of detection. To improve this detection level, bioimpedance cytometry can be used to independently characterize single cells and particles at a high flow rate [9,10]. This method is mainly focused on the characterization of physiological samples, such as blood cells, but it can be extended for other applications such as water quality analysis. Possible applications could be the detection of pathogenic bacteria and microalgae analysis. This non-invasive method does not require any biological or electrical markers and is particularly suitable for the integration in lab-on-a-chip sensors. Frequencies from several kHz to tens of MHz are typically used to perform single cell characterization. Measurements performed from lower to higher frequencies are able to characterize the electric and dielectric properties of media to the membrane and cell cytoplasm, respectively. Both the frequency band and impedance levels of a bio-sample increase with the size decrease of both eukaryotic and prokaryotic cells. It is necessary with this method to develop sensors with a sufficient bandwidth to characterize all cell parameters, as presented in References [11,12]. They developed sensors that were able to differentiate between different populations of cells up to 30 MHz and up to 500 MHz in physiological media using differential measurement configuration. Other impedance measurements at RF frequencies (several GHz to several tens of GHz) are also presented in Chien and Niknejad [13] and are generally limited to narrowband measurements. They were able to characterize other properties of biosamples, such as γ dispersion, mainly due to the electric dipole moment of water molecules. 

This paper is based on previous works [14,15], which had already demonstrated the ability of the first-generation sensor to detect and discriminate single cells at high rates (up to several thousand cells per second). We propose here a simple method to enhance the frequency band for cytometry sensors using bio-impedance characterization for analysis in low conductivity media. It appears in the review works of Petchakup et al. [9] that only a recent work [16] was performed on bacteria/yeast cells at lower conductivity media (0.1 S/m) and at frequencies above 1 MHz using a differential measurement method. It clearly appears that the development of cytometry sensors for the characterization of bacteria/cells in low conductivity media seems to be a promising area of research. 

In the case of analysis in low conductivity media analysis, such as water analysis, the impedance level can increase by up to a factor of 30 compared to physiological liquids. In these cases, parasitic coupling capacitances of the sensor can short-circuit the impedance of the sample at the higher frequencies and therefore should be taken into account when designing sensors. We also propose a method to fully characterize cytometry sensors, as well as their abilities and limitations, in order to perform true impedance measurements. Numerous works, as cited above, are based on the measurement of a differential voltage/current variation. Some of these studies do not characterize the sensor, its electrical model and limitations, and the true impedance of the sample. In these conditions, it is possible to differentiate two different populations of cells with the same sensor, but it is impossible to extract an intrinsic signature for each cell or compare measurements performed with two different sensors. In the case of a differential measurement and without cells (the only information is no presence of cells), the signal is null. In the case of a true impedance measurement, the system gives the impedance of the medium for the same measure. Medium conductivity can be calculated and is needed to extract all intrinsic parameters other than cell size, as presented in the next section.

The theoretical part of this paper presents a complete modeling of cytometry sensors, including all coupling capacitances. The next section shows the results obtained by the simulation of our first sensor design compared to the new one. In the fourth section, sensor fabrication is summarized and measurement protocols are described in detail. Results obtained for both generations of sensors were compared and discussed in the last two sections. 

## 2. Theory for Single Cell Characterization

### 2.1. Single-Cell Electrical Modeling

Electrical properties of biological samples and tissues can be described using equivalent electrical circuits and mathematical models. Fricke’s equivalent electrical circuit and Maxwell mixture theory (MMT) are usually combined to model macroscopic biological tissues [17].

The MMT model is well-adapted to describe a macroscopic and heterogeneous sample with a homogeneous repartition of its microscopic constituents. In the case of a single cell or particle, MMT must be adapted, as proposed by Morgan et al. [18], and is described in Equations (1)–(4). *C_med_* and *R_med_*, respectively, represent the dielectric and electrical properties of the medium, such as plasma in the case of a blood sample or water in the case of bacteria. *C_mem_* represents the dielectric property of the membrane, which is a nanometric bi-lipid layer. *R_i_* corresponds to electrical properties of the cytoplasm. *σ_med_* and *σ_i_* are the electrical conductivity in S/m of the medium and cytoplasm, respectively; *ε_med_* is the electrical permittivity of the medium in F/m and *C_mem,S_* is the surface capacitance of the membrane in F/m^2^. This surface capacitance can be approximated by using the equation of a parallel capacitor with the thickness of the membrane (several nm) and the relative permittivity of fat (around 5) to obtain a surface capacitance of approximately 1 µF/cm^2^. The parameter *k* is a geometrical factor that only depends on the geometry of the sample and on the cell/sample volume ratio *ϕ*. In the case of a single cell centered in a cubic volume, *k* corresponds to the size of one side of the reference cube. The parameter *r* is the radius of the cell.

(1) $$R_{med}=\frac{1}{\sigma_{med}\left(1-3\emptyset /2\right)k}$$

(2) $$C_{med}=\varepsilon_{med}\left(1-3\emptyset /2\right)k$$

(3) $$C_{mem}=\frac{9\emptyset rC_{mem,S}}{4}k$$

(4) $$R_{i}=\frac{4\left(\frac{1}{2\sigma_{med}}+\frac{1}{\sigma_{i}}\right)}{9\emptyset k}$$

According to Equations (1)–(4), it is necessary to make measurements at sufficiently high frequencies to characterize the internal properties of cells. This requirement is due to the high impedance of *C_mem_* at low frequencies. It is possible to determinate the cut-off frequency *F_c_* as a function of the coupled *C_mem_* and *R_i_*, as given in Equation (5). This frequency depends only on the nature of the sample (medium/cytoplasm conductivity and particle sizes) and is independent of the global sample geometry and the cell concentration.

(5) $$F_{c}=\frac{1}{2\pi R_{i}C_{mem}}$$

**Figure 1:**
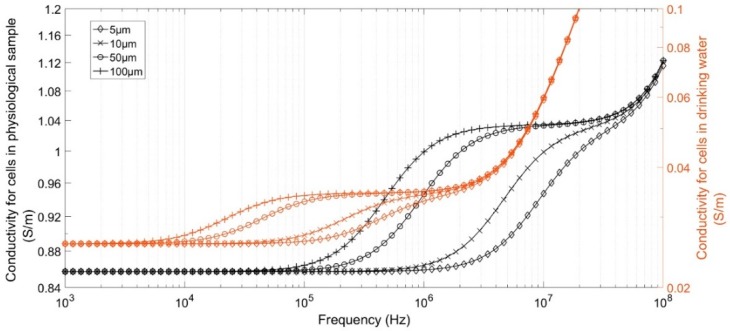
Complex conductivity module of samples versus cell diameter.

To study the impact of the medium conductivity and cell size on the value of this cut-off frequency and impedance, the modules of complex conductivity of four different cell sizes were computed using MMT equations. Cells sizes were chosen from 5 to 100 µm in diameter, corresponding to classical sizes of animal and plant cells. Two cases were tested to describe cells in a physiological medium, based on red blood cell properties, and cells/bacteria in drinking water, based on yeast cell properties [19]. Parameters are given in Table 1. Results are shown in Figure 1. The second plateau of curves corresponds to the impact of cytoplasm conductivity and demonstrate the necessity of performing measurements up to several tens of mega-Hertz to characterize the smallest cells. In media with lower conductivity, the cytoplasm properties could be detected at lower frequencies but with significantly higher impedance, making these more sensitive to coupling capacitance for impedance measurements. 

### 2.2. Sensor Structure

**Figure 2:**
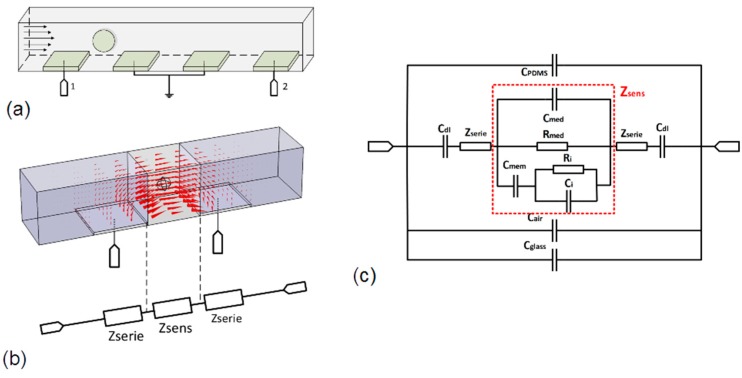
(**a**) Schematic of the microchannel sensing area, (**b**) schematic of current flow in the sensing area and equivalent electric model, and (**c**) equivalent electric circuit of cytometry microsensor with sample.

The sensing element was composed of a microchannel with a square section of the same order of magnitude as the cell sizes. The element was able to isolate, center, and move cells. Measurement was performed using two pairs of 20 µm × 20 µm coplanar electrodes placed along the channel, as shown in Figure 2a. This configuration permitted the monitoring of particle speeds using successive detections between these two pairs of electrodes and to facilitate the manufacturing process by placing all electrodes in the same layer. Useful measurements only corresponded to the maximum impedance variations when cells/particles were perfectly centered between electrodes. The channel was 20 µm wide, 20 µm high, and 1 mm in length to ensure the correct centering of cells and particles. This configuration was optimized for cells and bacteria characterization with diameters from a few µm to 15 µm and has already been discussed in previous works [20].

In the case of measurement in microchannels, the model described above can be difficult to apply. It is challenging to define the exact volume of the sensing area as well as the ratio *ϕ*. One can see a simulation of the current flow inside the channel in Figure 2b and the complexity to define only one reference volume. To solve this problem, the investigated volume was divided into three parts. The central part was a cubic reference volume centered in the measurement area and the other parts were two areas in series with it. Thus, the equivalent circuit corresponded to three impedances in series. As demonstrated by previous works [19], only *Z_sens_* is impacted when a cell is centered between electrodes. *Z_serie_* depends only on extracellular properties when the cell is centered between electrodes and can be calculated or simulated as a function of the complexity of the sensing structure. This model remains effective for cells/particles having a diameter between 30% and 70% of the channel width. The interest of this method is to model a single cell impedance in a simple cubic volume regardless of the electrode’s geometry and placement. It is thus possible to compare measurements performed with different cytometry devices.

### 2.3. Global Sensor Modeling

To complete the sensor model, the effect of coupling capacitances introduced by the electrical permittivity of used materials must be added. For macroscopic impedance measurements, these properties can be neglected in many cases when compared to the sample impedance. In our case, the volume of the samples was a picoliter. They present a high impedance, with a similar scale compared to coupling capacitances at megahertz frequencies. They act as a global equivalent capacitor in parallel with the impedance of the sample and can significantly reduce the frequency band by short-circuiting the impedance of the sample. It is essential to add these effects in the global model to optimize the sensor frequency band. Our sensors are fabricated with glass substrate and channels molded in polydimethylsiloxane (PDMS). It is possible to model these materials using three capacitors in parallel with the previous model, as shown in Figure 2c. These capacitors represent the effect of electrical permittivity of the glass substrates, PDMS channel, and air, respectively. They are proportional to the dielectric permittivity of materials and depend on electrodes’ geometries and thicknesses of materials. These factors can be difficult to model and are generally determined via simulation or measurement. 

*C_dl_* is the double-layer capacitance, which corresponds to an electrochemical phenomenon at the extreme surface of electrodes and acts as a barrier at low frequencies (Figure 2). The electrical resistance of platinum tracks is generally neglected at the microscopic scale due to the high impedance of samples.

## 3. Simulation of Optimized Structure

All simulations were performed with a finite element method (FEM) using COMSOL (Multiphysics^®^ COMSOL AB, Stockholm, Sweden) and the details about simulated models are given in Appendix A.

### 3.1. Simulation of the Sensing Area

**Figure A1:**
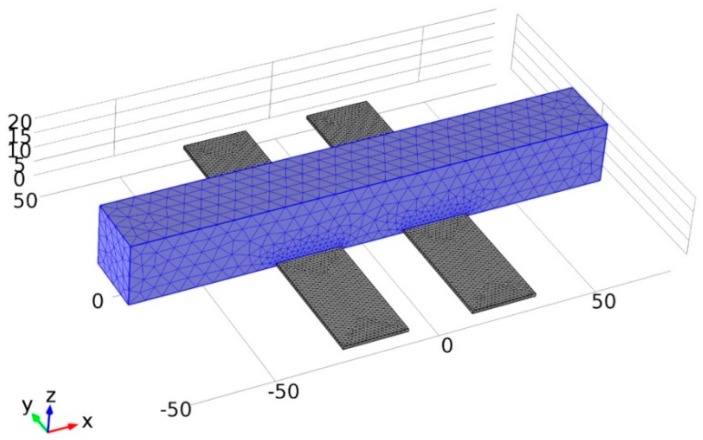
Simulated structure with coplanar electrodes and microchannel (scale in µm).

First, simulations were focused on the reference measurement area with no consideration of the capacitive effects of all other parts of the sensor. It was composed of 20 µm × 20 µm electrodes, with a width of 20 µm placed in a 20 µm × 20 µm section microchannel. Only electrodes and channels were studied to determine the geometrical parameter of the sensing area, as presented in Figure A1 in Appendix A. The simulation was performed by choosing an electrical conductivity of 1 S/m (physiological liquid) and 30 mS/m (tap water) with a relative dielectric constant of 78 for liquid samples without cells. Results are shown in Table 2. *R_sens_* and *C_sens_* were calculated using a geometrical factor of 20 µm corresponding to the cubic reference volume. *R_serie_* and *C_serie_* and were deduced using the model already presented in Figure 2b. As *Z_serie_* depends only on permittivity and conductivity of the sample, it can be modeled using Equations (1) and (2) with *ϕ* = 0 and a geometrical factor of 49.6 µm, deducted from simulations.

### 3.2. Sensor Optimization

**Figure 3:**
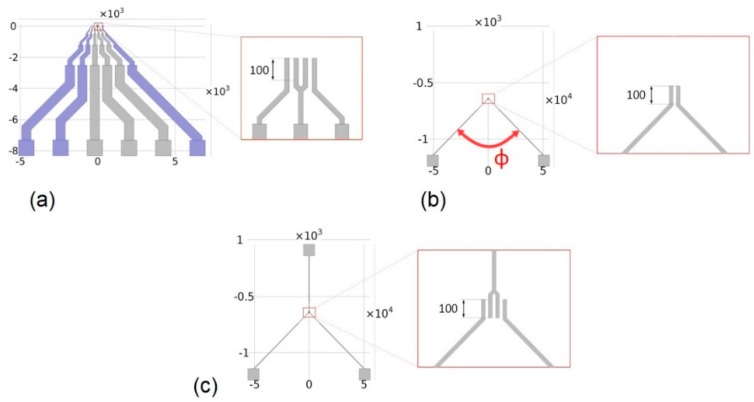
(**a**) Tracks design of the first sensor generation, (**b**) tested design, and (**c**) final design selected for the second sensor generation (scale in µm).

Most of the works on cytometry devices are focused on the description, modeling, and simulation of the sensing area, as presented in the previous section. To be connectable/pluggable into precision instruments and/or circuits, macroscopic tracks and pads must be added. Their design and impact are often neglected, even if the capacitive effect they induce is not negligible at higher frequencies. This capacitive effect was present regarding our first sensor generation, as shown in Figure 3a. It was designed as a printed circuit board (PCB) using high-width and parallel tracks with 45° angles. This configuration increased parasitic capacitances. Indeed, capacitance was proportional to track length, and width, and inversely proportional to track gap. To decrease the coupling capacitances, the width of tracks must be reduced and maximally spaced away from each other. 

**Figure 4:**
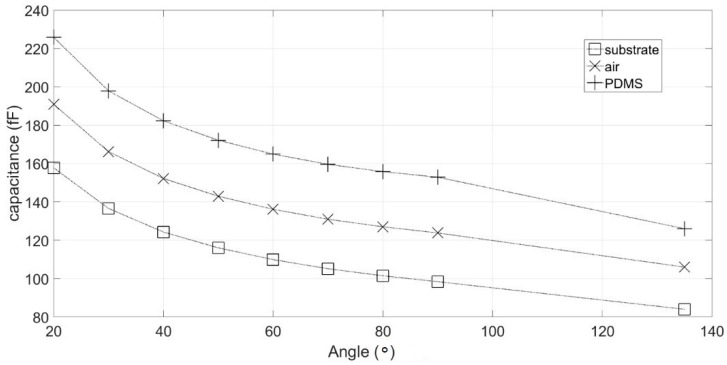
Simulated sensor capacitance as a function of electrodes angles and material.

**Figure A2:**
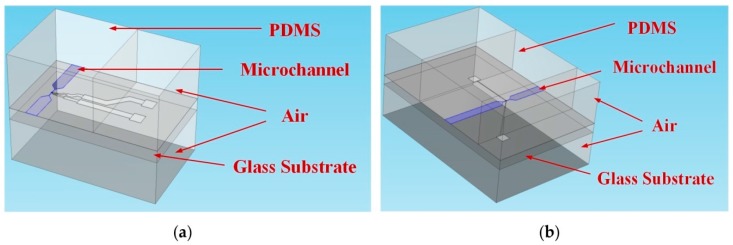
Simulated electrode structures: (**a**) first-generation sensor and (**b**) second-generation sensor.

To study the impact of tracks and materials, simulations were performed using a pair of electrodes from our previous sensor design as a reference. To optimize the track design, we propose the structure given in Figure 3b. It had a reduced track width (100 µm) with a length as short as possible, and the final pads remained the same. The impact of angle *ϕ* between tracks were studied via simulation. Results are presented in Figure 4. The three curves correspond to the capacitance induced by the substrate, the couple substrate/air, and substrate/air/PDMS combined, respectively. The simulated model is presented in Figure A2 in Appendix A.

It clearly appears that the global coupling capacitance was mainly caused by the dielectric properties of the substrate. Thus, the choice of substrate material was crucial. Glass seemed to be the best choice with its lower electrical permittivity compared to silicon (5 for glass versus 11.58 for silicon), higher electrical resistivity, and transparency to monitor samples. Capacitance with our previous sensor for the three-materials configuration were 305 fF, 377 fF, and 447 fF, respectively. For all angle configurations, the induced capacitance was at least 2 times lower than the capacitance induced by our previous generation. Furthermore, global capacitance was reduced by a factor of more than 3.5 in the best case. In accordance with this result, the reference electrode was placed in our new sensor design on the opposite side to the measurement electrodes with an angle of 135°. This new design is presented in Figure 3c.

## 4. Materials and Methods

### 4.1. Sensor Fabrication

**Figure 5:**
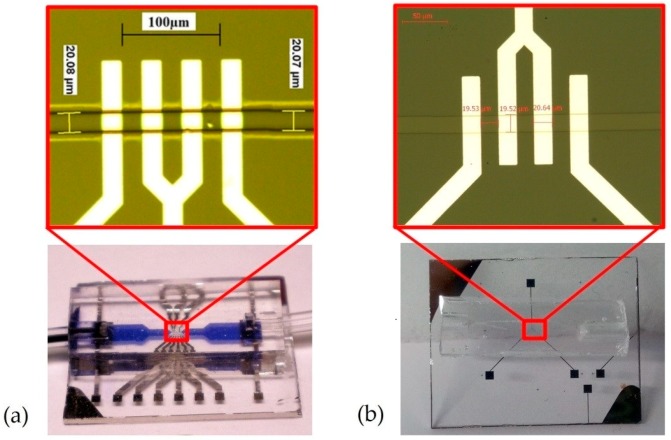
Photographs of (**a**) the first-generation sensor and (**b**) the second-generation sensor.

Sensors were fabricated in a clean room using a standard optical lithography process and biocompatible materials such as glass substrates, platinum electrodes, and a PDMS channel. Platinum electrodes were deposited on the glass substrate using sputtering, structured by exposing a lithography resin to UV beams and ion beam etching (IBE). The channel was structured by molding PDMS on a negative SU-8 resin mask and glued to the substrate with 02-plasma treatment. A photograph of the two generations of sensors is given in Figure 5.

### 4.2. Measurement Setup

**Figure A3:**
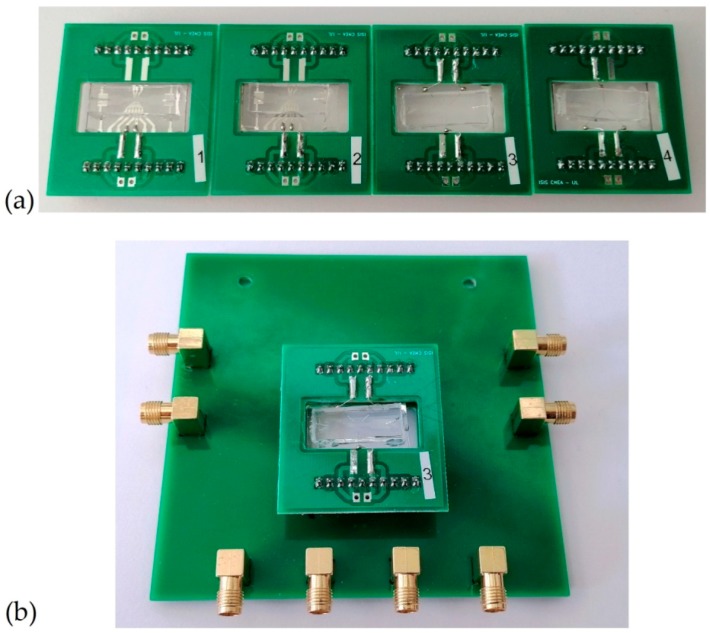
(**a**) Photograph of the cytometry biosensors connected on an individually numbered PCB. Sensors S1–S2 were first generation and S3–S4 were second generation. (**b**) Photographs of a sensor plugged into a test plate.

To perform reproducible tests, four sensors (two of which were first generation and the remaining two being second generation) were placed on identical small printed circuit boards (PCBs), as described in the Figure A3 in Appendix B. 

The first measurement was performed in static conditions (without flow into the channel) using a precision impedance analyzer (Keysight E4990A, Keysight Technologies, Santa Rosa, CA, USA). This instrument is able to perform particularly high accuracy and wide impedance measurements in the range of 25 mΩ to 40 MΩ using an auto-balancing bridge method (four-point probe method). Results obtained in the “No-Load” configuration (without liquid samples) and the measurement of the characteristic impedance of the sample medium will be used as a reference for the next step described below. 

**Figure A4:**
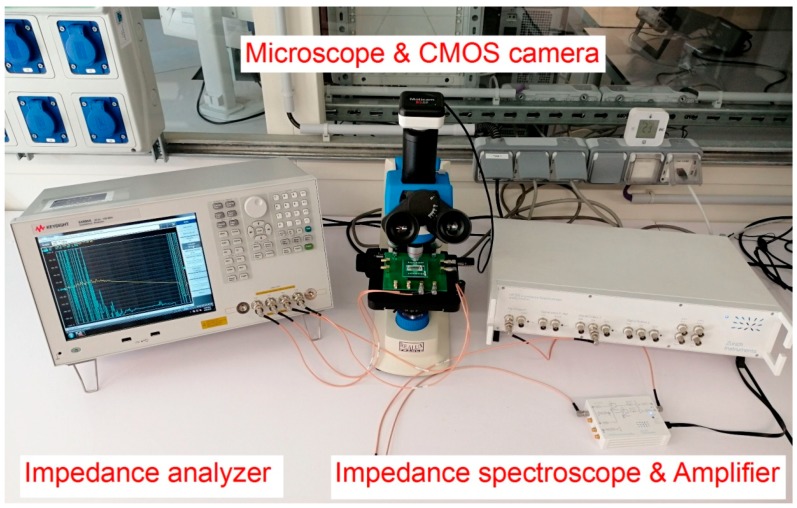
Photographs of the measurement setup with impedance analyzers, impedance spectroscope with amplifiers, and optical microscope with sensors.

The second measurement was performed in dynamic conditions (with flow) using a HF2IS Impedance Spectroscope (Zurich Instruments). This spectroscope, based on the “lock-in amplifier” principle, permits the measurement of multi-frequency impedance measurements (up to four from 1 µHz to 50 MHz) at a particularly high rate (up to tens of thousands impedances/s). According to the high impedance of our system, in the scale of MΩ, a two-points configuration was chosen as recommended by the manufacturer. In this configuration, the voltage excitation signal was applied at a terminal and the current was measured at the other terminal using a HF2TA Current Amplifier (Zurich Instruments). The high transimpedance gain necessary for our application (10 kV/A) limited the bandwidth to 8 MHz. A photograph of the measurement setup is given in Figure A4 in Appendix B.

During all the measurements, the liquid sample injection and its flow into the microchannel was monitored using an optical microscope coupled to a Complementary Metal Oxide Semiconductor CMOS camera. A computer was used to command the devices and the measurement was recorded using LabVIEW’s programs (National Instruments, Austin, TX, USA). An image of the measurement setup is shown in Figure A4 in Appendix B. All measurements presented in this paper were performed at room temperature (23 °C ± 1 °C).

### 4.3. Sample Choice and Preparation

The microfluidic sensors were designed to detect and characterize living cells and particles in liquid samples, with a typical size of a few µm to 15 µm (diameter). To demonstrate the good ability of the new-generation sensors for analysis in low conductivity media, tap water was chosen as a basic sample. Drinking water with an average concentration of minerals, such as tap or spring water, present an electrical conductivity of approximately 30–50 mS/m. This figure is about 30 times lower than the conductivity of physiological liquids, such as blood plasma. This lower conductivity increases the impedance of the sample, and at the same time, it is difficult to perform measurements at higher frequencies due to the effect of parasitic capacitances. 

Half a liter of tap water was taken and de-chlorinated using a carbon filter. A typical conductivity of 33 mS/m at 24 °C was measured using a Keysight 16452A liquid test fixture with the impedance analyzer. 

The biological model selected for the tests was non-purified yeast cells (*Saccharomyces cerevisiae*). This organism was chosen because it is non-toxic, easily manipulated, and comprises simple cells, serving as a model for all eukaryotes. It is also resistant to a large range of conductive media from tap water to physiological media without lysis effects. Dry cells were diluted in reference water to obtain a concentrate sample, and then was diluted several times to produce a low concentration (less than 1% in volume) to avoid channel locking. The aim of the presented work was to demonstrate the ability to measure all of the electrical properties of a biological cell (mainly cytoplasm conductivity); the purity and exact concentration of cells being analyzed were not critical. 

All samples were injected into the microchannel using a syringe. Measures were recorded after air and liquid flowed for at least ten seconds to clean the microchannel and limit possible contamination caused by previous samples. 

## 5. Results

### 5.1. Sensor Characterization

These results concern the measurements performed without a sample and with the reference water sample to study the overall behavior of each generation of sensors. Measurements were performed across the full frequency range of the spectrometer to highlight abilities as well as limitations. 

**Figure 6:**
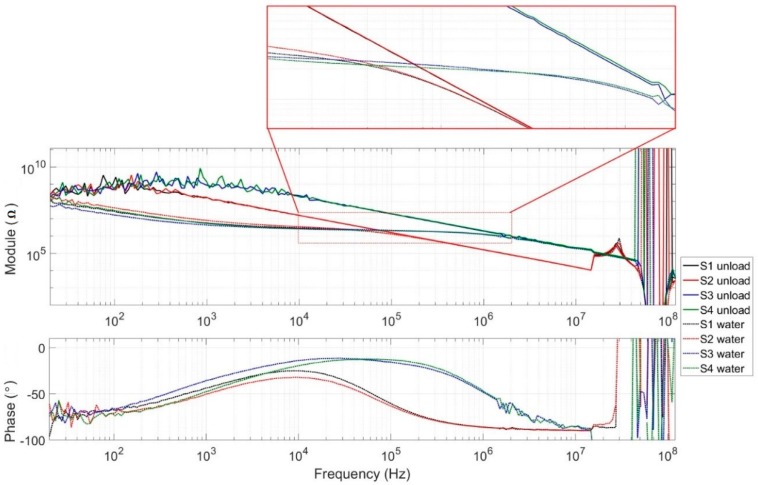
Measured impedance module and phase of the new and the previous generations of unloaded sensors.

Results without samples are presented in the form of a Bode diagram in impedance (module) in Figure 6. We can see a global capacitive comportment without samples for all sensors. In the lower frequencies, a noise phenomenon appeared, which was due to the limits of the spectrometer and was observed when the impedance was higher than 40 MΩ. A resonance phenomenon was present at the higher frequencies and was similar to both new and previous-generation sensors. This phenomenon can be attributed to the connection, cable, and test board, and limited the bandwidth to 15 MHz. For that reason, the next result spectrum will be limited to 15 MHz.

The new sensors (labeled S3 and S4) presented a significantly lower coupling capacitance than the previous generation (named S1 and S2). The S3 and S4 sensors had an empty capacitance of 82 fF ± 5% compared to 995 fF ± 0.5% for S1 and S2. This is a factor ratio higher than 10 between the two sensors. These results are on the same scale as the simulation results but still present a significant shift. These differences could be explained regarding the new sensor by the difference of the scales between microscopic width and macroscopic length of the electrodes’ tracks that could induce approximations with FEM simulations. For the previous sensor, only one pair of electrodes was simulated to reduce the time of the simulation. However, other tracks on each side of the studied electrodes could add coupling capacitances. 

The second set of results, presented in Figure 6, were obtained by injecting reference water into the microchannel of each sensor. For all sensors, curves seemed to follow an asymptote corresponding to curves obtained without a sample. All curves presented a plateau at the same level corresponding to the electric conductivity of reference water. 

One can observe a decreasing impedance over several hundreds of kHz for sensors S1 and S2 compared to the new sensors, which presented a frequency band with a little more than one decade. According to the phase measurement, the capacitive effect became predominant over 1 MHz for the first generation compared to 10 MHz for the second generation. As presented in Section 2, small cells such as yeast or bacteria, require measurement up to several MHz to be completely characterized (characterization of the cytoplasm) and the new sensor appeared to be more capable of performing such a measurement. 

### 5.2. Static Yeast Cell Characterization

**Figure A5:**
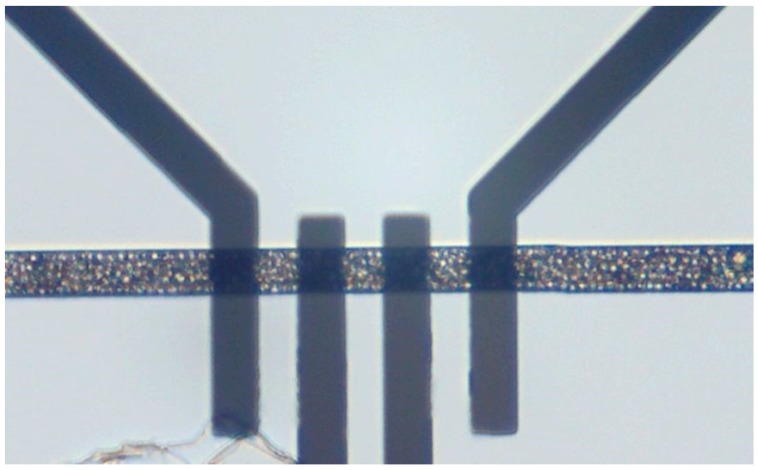
Examples of the yeast cap obtained in the measurement area via the injection of a sample with high cell concentration for sensor S4 (magnification 100×).

To ensure the capability of our new sensors to characterize yeast cells, it was necessary to determine whether the electric effect of the cytoplasm could be detected. Static characterizations of yeast cells were performed for both generations of sensors. Because of the very small size of the measurement area, it was nearly impossible to place and maintain a cell rigorously centered between electrodes during the acquisition time of approximately 1 min. To remedy this issue, a sample with a high concentration of cells was injected to create a cap and immobilize cells into the measurement area, as shown in Figure A5 in Appendix B. Even if the conditions were clearly different from the passage of just one cell, key frequencies stayed the same irrespective of the concentration, as discussed in Section 2.

**Figure 7:**
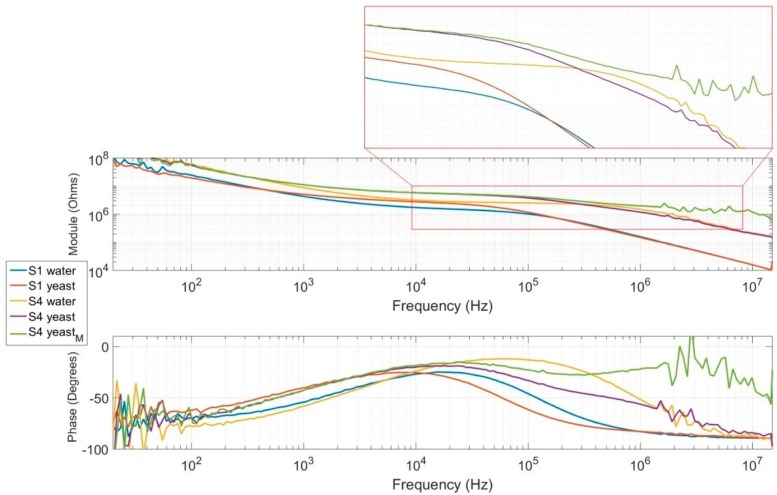
Measured impedance module and phase of new (S4) and previous-generation (S1) sensors with a yeast cell cap on the measurement area.

Only one sensor of each generation (S1 and S4) was used for this test because the cap was impossible to clear. Measurements were performed with and without the cap, and results are presented in Figure 7. S4_yeastM_ corresponds to the application of measurement compensation on S4_yeast_. The compensation consisted of removing the impedance of the sensor measured without any sample via calculation. The “water” curve corresponds to measurements performed with the same sample but without caps and cells between electrodes. For both sensors, measurements without a cap presented the same type of spectrum as the reference water. When the cell cap was present, the level of the plateau increased in medium frequencies by around 10 to 100 kHz. At these frequencies, cells could be considered insulated and limited current flow. At higher frequencies of approximately 1 MHz, a second plateau appeared in the diagram of sensor S4, corresponding to the contribution of the cell’s cytoplasm and provided strong evidence for our assumptions. This resulted in the presence of a second dome in the phase diagram, clearly visible in the green curve. The slope between these plateaus represents the capacitive effect of the cell membrane. This second plateau is not present in the diagram of the previous sensor generation because of its smaller bandwidth compared to the new design. 

### 5.3. Yeast Cell Cytometry

Final measurements were performed in dynamic condition during the passage of single yeast cells in the measurement area. The aim of these tests was to prove the ability of our new sensor to measure the effect of cell cytoplasm for single yeast cells. This effect appeared at the higher measurement frequency (our operating frequency band remained below 100 MHz). As described in the previous section, a HF2IS impedance spectroscope was used with a multi-frequency excitation signal. All performed measurements provided true impedance (module and phase) and not relative impedance. According to previous results, 100 kHz and 1 MHz frequencies were selected with an amplitude of 250 mV and at 7200 impedance measurements per second. The first frequency corresponds to the frequency where the resistive effect was at a maximum of the reference liquid. The second frequency corresponds to the more suitable frequency to use to detect the second plateau. Measurements were performed using sensors S2 and S3 in the same conditions and with the same sample, i.e., a dilution of yeast cells with a concentration of less than 1%. 

**Figure 8:**
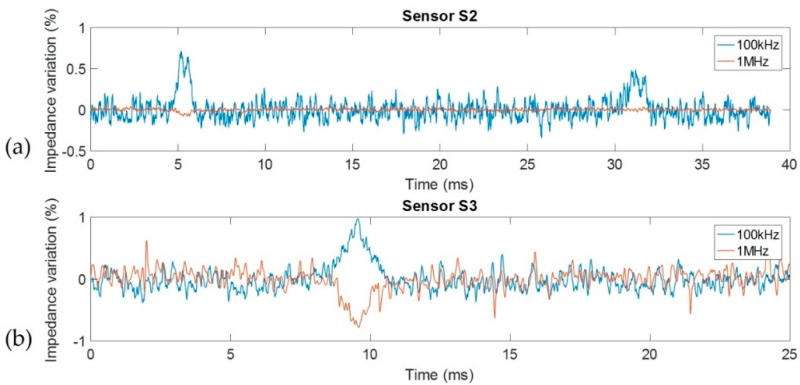
Impedance variation for 100 kHz and 1 MHz frequencies (in percent) during the passage of yeast cells with (**a**) first-generation sensor S1 and (**b**) second-generation sensor S4.

Results, presented in Figure 8, are shown as the impedance (module) variation as a function of time for both 100 kHz and 1 MHz fixed frequencies. This representation is able to clearly show the impact of cell volume at low frequency and the impact of cytoplasm conductivity at higher frequency. For both sensors, the variations observed at 100 kHz were similar and demonstrate the ability to detect the passage of cells and determine their sizes. Each brief increase of impedance corresponds to the passage of a single cell. Thus, the cell volume is directly proportional to impedance variation. We can notice the ability to measure particularly small impedance variations around 0.5%. Observed noise can be attributed to both the measurement setup and to the ionic noise of the sample. The impedance of ionic solutions is highly sensitive to pressure and temperature: a variation of 1 °C caused a variation of the impedance of around 2%. At 1 MHz, impedance did not present significant variation during the passage of cells for the previous-generation sensor. In this case, capacitive effects were predominant and short-circuited the effect induced by the presence of a sample, reducing the noise level at the same time. For the second-generation sensor, capacitive effects were not yet predominant, and the effect of cell passage was clearly visible. This time, cell presence induced a decrease of impedance in the same level order of magnitude as the increase observed at 100 kHz, and demonstrates the contribution of the cytoplasm conductivity in global impedance. The cytoplasm had a characteristic conductivity of around 0.25 S/m, approximately 10 times higher than the medium conductivity. Therefore, yeast cells can be considered a particularly effective electrical conductor face to the medium at 1 MHz and induced the opposite variation than at 100 kHz. 

Using true impedance and our model described in Figure 2, simulation results and Equations (1)–(4), it is possible to analytically extract cell size, cytoplasm conductivity, and membrane capacitance directly from measurements. In general, a system based on differential measurement and/or without an adapted model first needed a calibration with calibrated beads as a reference to extract some parameter from the cell measurement. Calibrated beads are interesting to validate the performance of sensors. Cell size and cytoplasm conductivity can be obtained by measuring resistance variations at 100 kHz and 1 MHz, respectively. Determination of the membrane capacity needed another measurement at a medium frequency (approximately 300 kHz), where the capacitive effect is predominant. Using the variations obtained in Figure 8b, around 0.9% for 100 kHz and −0.9% for 1 MHz, we calculated a cell size of 2.73 µm radius with a cytoplasm conductivity of 137 mS/m. (Details calculation are given in Supplementary Materials). These results are in accordance with the classical properties of yeast cells. 

Since the first-generation sensor is unable to characterize cytoplasm properties, these results prove the necessity of optimizing the sensor in the case of measurement in low conductivity media. 

## 6. Discussion and Conclusions

A method to optimize the frequency band of a cytometry biosensor for low conductivity media analysis was described. Applications of low conductivity media analysis could cover the detection of pathogenic bacteria and microalgae analysis, and seems to be a promising area of research. Based on a complete electric modeling of a cytometry sensor with biological samples, a theoretical study demonstrated the necessity to take into account and optimize the electrodes and tracks placement in the case of a high impedance sample analysis. These placements could produce a non-negligible capacitive effect sufficient to short circuit measurements. Simulations performed using FEM permitted us to validate a new optimized design by comparison with our first-generation sensor. Results have demonstrated the possibility to significantly expand the frequency bandwidth by at least a factor of 5 by only optimizing the electric tracks’ placement and geometries without modifying the design of the sensing area. After the fabrication of the new-generation sensors, measurements performed without samples and with reference water demonstrated an increase in the frequency bandwidth of more than one decade compared to the previous generation. The inability of our first non-optimized sensor to characterize all yeast cells properties proved the necessity to optimize sensors for measurements in low-conductivity media. Tests performed in static and dynamic conditions with yeast cells provided evidence for the possibility to fully characterize cells with our new sensors. The method was able to better detect single cells and measure their sizes, as well as the electrical properties of the membrane and the cytoplasm, compared to the previous generation, which was only able to detect cells and measure their sizes. 

## Figures and Tables

**Table 1:** Parameters used for samples conductivity calculation.

Type of Sample	*σ_med_*	*ε_med_*	*σ_i_*	*ε_i_*	*C_mem,S_*	Volume Ratio *ϕ*
Cells in physiological media	1	78	1.5	78	1 µF/cm^2^	0.25
Cells in drinking water	0.03	78	0.25	50	1 µF/cm^2^	0.25

**Table 2:** Simulated capacitance (in fF) for first- and second-generation sensors.

Configuration	*R_mes_* (kΩ)	*C_mes_* (fF)	*R_sens_* (kΩ)	*C_sens_* (fF)	*R_serie_* (kΩ)	*C_serie_* (fF)	*Z_sens,factor_* (µm)	*Z_serie,factor_* (µm)
*σ* = 1 S/m *ε_r_* = 78	90.28	7.65	50	13.81	20.14	33.85	20	49.65
*σ* = 30 mS/m *ε_r_* = 78	3009	7.65	1666	13.81	672	33.85	20	49.6

## Supplementary Materials

The detailed calculation of cell parameters are available online at https://www.mdpi.com/1424-8220/19/15/3366/s1.

## Appendix A

All simulations were performed with a finite element method (FEM) using COMSOL Multiphysics^®^ to compare the performance of our previous sensor design to the new optimized design. Both designs present the same sensing area composed of 20 μm × 20 µm electrodes with a width of 20 µm placed in a 20 µm × 20 µm section microchannel. Figure A1 presents the meshing used for the simulation of the sensing area composed of platinum electrodes and a channel full of water with 1 S/m conductivity.

To simulate the capacitive effect of connection tracks, one pair of electrodes without a sample was simulated for each sensor structure, as presented in Figure A2. Simulations were performed first only with a glass substrate, the second with a glass substrate and air on each side of the sensor, and finally by adding a 1 mm in width and 5 mm in height PDMS block to study the impact of each material.

## Appendix B

Figure A3a shows the four sensors (S1 to S4) used to perform tests. They were soldered to a small PCB piece with 1/10-inch System-in-Package (SIP) connectors. With this configuration, it is easy and quick to change sensors from the test board presented in Figure A3b. The test board was a PCB with SIP connectors to plug in sensors and SMA connectors for instrument connection with two- or four-point measurement capability. This test board could be plugged into an optical microscope for monitoring purposes. Photographs of the measurement setup is shown in Figure A4.
