# Supplementary material for: Lab-On-A-Chip Device for Yeast Cell Characterization in Low-Conductivity Media Combining Cytometry and Bio-Impedance
Source: Sensors (Basel). 2019 Jul 31;19(15):3366. doi: 10.3390/s19153366 (PMC6695822; doi:10.3390/s19153366)
Supplement: Supplementary file 1 [file sensors-19-03366-s001.pdf]

sensors

Detailed Calculation of Cell Parameters

This file gives complementary information about the calculation of cell properties in function as measured impedance.

- $R_{mes,ref}$ represents the measured resistance when no cell is presents between electrodes.
- $R_{sens,ref}$ represents the measured resistance of sensing area (cubic reference volume) when no cell is presents between electrodes.
- $R_{meas,cell}$ represents the measured resistance when a cell is centered between electrodes.
- $R_{sens,cell}$ represents the measured resistance of sensing area (cubic reference volume) when a cell is centered between electrodes
- $\Delta R_{meas} = R_{meas,cell} - R_{meas,ref}$
- $\Delta R_{sens} = R_{meas,cell} - R_{meas,ref}$

The other variables used in this document are the same than variables described in the article.

1. Calculation of Cell Size

At the lower measurement frequencies, cell can be considered as insulating and cell size can be calculated as followed:

- $R_{meas,cell} = 2R_{serie} + R_{sens,cell} = 2\frac{1}{\sigma_{med} k_{Z,serie}} + \frac{1}{\sigma_{med}(1-3\emptyset/2)k_{Z,sens}} = \frac{1}{\sigma_{med}}\left(\frac{2}{k_{Z,serie}} + \frac{1}{(1-3\emptyset/2)k_{Z,sens}}\right)$
- $\frac{\Delta R_{meas}}{R_{meas,ref}} = \frac{\frac{1}{\sigma_{med}}\left(\frac{1}{(1-3\emptyset/2)k_{Z,sens}} - \frac{1}{k_{Z,sens}}\right)}{\frac{1}{\sigma_{med}}\left(\frac{2}{k_{Z,serie}} + \frac{1}{k_{Z,sens}}\right)} = \frac{\frac{1}{k_{Z,sens}}\left(\frac{1}{(1-3\emptyset/2)} - 1\right)}{\left(\frac{2}{k_{Z,serie}} + \frac{1}{k_{Z,sens}}\right)} = \frac{\left(\frac{1}{(1-3\emptyset/2)} - 1\right)}{\left(\frac{2\,k_{Z,sens}}{k_{Z,serie}} + 1\right)}$
- $\left(\frac{1}{(1-3\emptyset/2)}\right) = \left(\frac{2\,k_{Z,sens}}{k_{Z,serie}} + 1\right)\frac{\Delta R_{meas}}{R_{mes,ref}} + 1$
- $(1-3\emptyset/2) = \frac{1}{\left(\frac{2\,k_{Z,sens}}{k_{Z,serie}} + 1\right)\frac{\Delta R_{meas}}{R_{mes,ref}} + 1}$
- $\emptyset = \frac{2}{3}\left(1 - \frac{1}{\left(\frac{2\,k_{Z,sens}}{k_{Z,serie}} + 1\right)\frac{\Delta R_{meas}}{R_{mes,ref}} + 1}\right)$

In the case of our sensor $k_{Z,sens} = 20 \times 10^{-6} m$ and $k_{Z,serie} = 49.6 \times 10^{-6} m$:

- $\emptyset = \frac{2}{3}\left(1 - \frac{1}{1.806\,\frac{\Delta R_{meas}}{R_{mes,ref}} + 1}\right)$

Example for $\frac{\Delta R_{meas}}{R_{mes,ref}} = 0.9\%$ at low frequency:

- $\emptyset = \frac{2}{3}\left(1 - \frac{1}{1.806 \times 0.009 + 1}\right) \approx 1.07 \times 10^{-2}$
- $Cell_{vol} = \emptyset\, Ref_{vol} = \emptyset\, {k_{Z,sens}}^{3} \approx 8.53 \times 10^{-17} = 85.3 \mu m^3$
- If we consider a spherical cell, we obtain a radius of 2.73 µm.

2. Calculation of Cytoplasm Conductivity

At the higher measurement frequency, cell membrane impedance can be neglected and cytoplasm conductivity can be calculated as followed:

- $R_{sens,ref} = \frac{1}{\sigma_{med} k_{Z,sens}}$ *and* $R_{serie} = \frac{1}{\sigma_{med} k_{Z,serie}}$ $\leftrightarrow$ $R_{serie} = R_{sens,ref} \frac{k_{Z,sens}}{k_{Z,se}}$
- $\frac{\Delta R_{meas}}{R_{meas,ref}} = \frac{R_{sens,cell} - R_{sens,ref}}{2R_{serie} + R_{sens,ref}} = \frac{R_{sens,cell} - R_{sens,ref}}{2R_{sens,ref} \frac{k_{Z,sens}}{k_{Z,se}} + R_{sens,ref}} = K \frac{R_{sens,cell} - R_{sens,ref}}{R_{sens,ref}}$ with $K = \frac{1}{2 \frac{k_{Z,sens}}{k_{Z,se} rie} + 1}$
- $\frac{R_{sens,cell} - R_{sens,ref}}{R_{sens,ref}} = \frac{\frac{R_i R_{med}}{R_i + R_{med}} - R_{sens,ref}}{R_{sens,ref}} = \frac{-R_{sens,ref}}{R_i + R_{sens,ref}} = \frac{1}{K} \frac{\Delta R_{meas}}{R_{meas,ref}}$
- $\frac{R_i R_{med}}{R_i + R_{med}} = R_{sens,ref} (1 + K_2)$ with $K_2 = \frac{1}{K} \frac{\Delta R_{meas}}{R_{meas,ref}}$

As $R_{sens,ref} = \frac{1}{\sigma_{med} k_{Z,sens}}$ and $R_{med} = \frac{1}{\sigma_{med} (1 - 3\emptyset/2) k_{Z,sens}}$ can be measured during cell passage or calculate after measuring medium properties, R_i can be easily calculate as followed:

- $\sigma_i = \frac{1}{\frac{9 \emptyset k_{Z,sens} R_i}{4} - \frac{1}{2\sigma_{med}}}$

Example for $\frac{\Delta R_{meas}}{R_{mes,ref}}$ = 0.9% at high frequency:

- $K_2 = \frac{1}{K} \frac{\Delta R_{meas}}{R_{meas,ref}}$ = 0.01626 = 1.626%
- $R_{sens,ref} = \frac{1}{0.033 \times 20 \times 10^{-6}} = 1{,}5152\ \text{M}\Omega$
- $R_{med} = \frac{1}{0.033 \times (1 - 3 \times 1.07 \times 10^{-2}) \times 20 \times 10^{-6}} = 1{,}5398\ \text{M}\Omega$
- $R_i = 46{,}62\ \text{M}\Omega$
- $\sigma_i = \frac{1}{\frac{9 \times 1.07 \times 10^{-2} \times 20 \times 10^{-6} \times 46{,}62 \times 10^{6}}{4} - \frac{1}{2 \times 0.033}} \approx 0{,}137\ \text{S/m}$

3. Calculation of Membrane Surface Capacitance

At medium frequency, cell membrane capacitance prevails face to other capacitance and can be easily measured. The membrane surface capacitance can be calculated using the following equation:

- $C_{mem,S} = C_{mem} \frac{4}{9\emptyset r k_{Z,sens}}$
